# Crowdsourcing architectural beauty: Online photo frequency predicts building aesthetic ratings

**DOI:** 10.1371/journal.pone.0194369

**Published:** 2018-07-25

**Authors:** Albert Saiz, Arianna Salazar, James Bernard

**Affiliations:** 1 Urban Studies and Planning Department, Massachusetts Institute of Technology, Cambridge, Massachusetts, United States of America; 2 Economics Department, Brown University, Providence, Rhode Island, United States of America; University of Warwick, UNITED KINGDOM

## Abstract

The aesthetic quality of the built environment is of paramount importance to the quality of life of an increasingly urbanizing population. However, a lack of data has hindered the development of comprehensive measures of perceived architectural beauty. In this paper, we demonstrate that the local frequency of geotagged photos posted by internet users in two photo-sharing websites strongly predict the beauty ratings of buildings. We conduct an independent beauty survey with respondents rating proprietary stock photos of 1,000 buildings across the United States. Buildings with higher ratings were found more likely to be geotagged with user-uploaded photos in both Google Maps and Flickr. This correlation also holds for the beauty rankings of raters who seldom upload materials to the internet. Objective architectural characteristics that predict higher average beauty ratings of buildings also positively covary with their internet photo frequency. These results validate the use of localized user-generated image uploads in photo-sharing sites to measure the aesthetic appeal of the urban environment in the study of architecture, real estate, urbanism, planning, and environmental psychology.

## Introduction

Our ability to understand the effect of a city’s environment on social outcomes has been limited by a lack of data on the physical perception of urban space, such as the subjective beauty of buildings, the appearance of neighborhoods, and the emotional cues that these convey. New data tools make it increasingly possible to measure these perceptual attributes, which may be useful for urban planners and scientists looking to explain the social consequences of urban development.

In this paper, we validate the use of large amounts of geotagged user-generated images to measure the average subjective beauty of buildings in a scalable, comparable, and systematic way. We use the geographic location of all images obtained from two different photo-sharing websites (Flickr^®^ and Panoramio^®^), and we estimate the frequency of photos posted in the vicinity of each building in a catalog of 206,216 properties in the United States. To validate online posting frequency as a metric for building beauty, we conducted a survey where 605 respondents ranked a subsample of 1,000 buildings according to their perceived beauty using separate professional stock photos. We show that all of our measures of localized internet image uploads correlate strongly and positively with building beauty. The relationship holds across sub-groups of respondents who have different demographic characteristics. Both image uploads and building beauty measurements display similarly-signed covariances with groups of observable building characteristics, such as height, age, and architectural style. Importantly, the conditional correlation between image uploads and building beauty vanishes for photos taken more than 50 meters from the building of interest, which negates alternative explanations based on spurious or contextual effects.

Our research informs a growing body of literature that explores the connection between social science, urban form, architecture, and city planning. Prior research has focused on how the built environment impacts outcomes such as the quality of life, psychological well-being, and social connections. For example, [1] hypothesized that urban design could influence the level of crime by creating “defensible space.” An extensive literature in environmental psychology has demonstrated strong connections between the visual appearance of a city’s neighborhoods and the behavior and health of its citizens (e.g., [2]). [3], for example, show that individuals exposed to more scenic environments report better health across urban, suburban, and rural areas. In fact, [4] find that models including crowdsourced data from Flickr and OpenStreetMap can generate more accurate estimates of “scenicness” than models that consider only basic census measurements. Our paper focuses on building-level characteristics and complements existing evidence that has focused on area-level attributes.

Efforts to quantify neighborhood appearance in architecture have used visual perception surveys, in which people were asked to rate or compare images. These methods have been used to create perceptual maps of cities ([5]; [6]; [7]). However, the manual nature of traditional data collection processes meant that they could not be deployed over large geographical areas or different time periods. Therefore, the recent data explosion can help advance our understanding of urban perception. Research by [8] shows how user ratings of online images can be combined with machine-learning methods to measure perceptions of street safety. Complementary research by [9] opens up further avenues for generating aesthetics estimates by using deep learning techniques to generate ratings for photographs of the built environment that survey participants have not previously rated.

A number of papers have previously used existing internet image geolocations to study patterns of city attractiveness. For example, [10] compare statistics of the spatial distribution of user-uploaded photos across ten cities, showing that European cities have more centralized patterns of photo uploads. [11] use unsupervised methods to extract representative views and images for landmarks. [12] show that tourists and native city-dwellers display similar photo uploading patterns and that tourists display strong seasonality, and [13] uses photo upload frequencies to identify high-amenity areas in central Berlin and London. Other researchers have analyzed user behavior on photo sharing services. [14], for example, identify a broad range of motivational factors that are associated with participation in online photo-sharing communities, and [15] show that web applications, such as Flickr, can serve as reliable, universal sources of spatial content.

Our research also complements papers that have used online user-generated content to extract time-series data about consumer behavior ([16]), health ([17]; [18]), or finance ([19]), or to obtain cross-sectional socioeconomic data ([20]). A growing literature in urban tomography ([21]) is demonstrating that adding geographical identification to such methods can improve research and practice in urban planning, urban sciences, environmental science or psychology, and architecture. For example, [22] shows the conditions under which user-generated opinions can be deemed reliable for planning decisions. [23] and [24] show how the local frequency of online Flickr photo tags –short texts accompanying the images– can be used ingeniously to describe relevant qualitative attributes of urban environments as perceived by posters.

Our results indicate that the upload frequency of images can be used to create scalable quantitative measures of aesthetic perception of specifically-targeted buildings. The localized frequency of image uploads provides a readily available beauty metric that can be useful for practitioners and researchers seeking to link urban perception with other social, political, economic, and cultural aspects of cities. Moreover, we also examine the relative impact of architectural styles on building beauty, thus informing our understanding of the urban environment.

The rest of the paper is organized as follows. In section 2 we construct the image upload measurements using two different photo-sharing websites. Section 3 turns to our building survey structure, building beauty measurements for the survey subsample, and the validation of image uploads as an alternate beauty proxy. Section 4 concludes and suggests future research directions and applications.

## Localized internet photo frequencies

### Measurement

We first obtained data from Flickr and Panoramio (the source of the images in Google Maps^®^ until November 4, 2016). These photo-sharing websites contain millions of geotagged photos contributed by people from all around the world. We used the Flickr Application Programming Interface (API) to download all of the approximately 13 million open-access photos that were geotagged by users at the maximum accuracy level in the United States between February 1, 2004, and January 1, 2014. The maximum accuracy corresponds to photos which already contain GPS coordinates from the camera or to those where the user has presumably zoomed into the relevant street in order to pin down the photo to its location. To ensure consistency of results, we followed a similar collection process for Panoramio at two separate points in time. We thus obtained information on approximately 800,000 photos posted in 2011 (prior to our survey) and approximately 3 million photos posted in 2014 (after we completed our survey but before the data analysis).

To count the number of photos in the vicinity of each building, we took the following steps. First, we combined both Panoramio and Flickr data with a proprietary dataset produced by Emporis^®^, which contains information on the exact location and characteristics (such as height, age, and architectural style) of 206,216 major buildings across the United States. Second, we used GIS software to assign the user-generated images to each building on the basis of the distance between their spatial location. Third, we counted the number of photos within each annuli (two-dimensional “donuts”) of different lengths around each building in our sample: starting at 0–50 meters, 50 to 100 meters, 100 to 250 meters, and ending at 250 to 500 meters. For robustness, we also counted the number of pictures using 10-meter intervals that started from 0 to 10 meters, and continued up to 490 to 500 meters. We refer to these several measures as “Flickr or Panoramio image uploads”.

Table 1 summarizes the image upload measures. We focus on the number of pictures taken within 50 meters of a building in Panoramio, which corresponds to the main distance used in the empirical analysis. Column 1 shows that on average, in 2011, there were 0.242 pictures taken within 50 meters of the buildings in this sample (standard deviation = 0.652). This number rose to 0.612 in 2014 (standard deviation = 4.15), which presumably reflects the increased access to technology and social media in recent years. As expected, the distribution is very skewed: 87 percent of the buildings had no image assignment in 2014. The Flickr data up until 2014 shows an average of 2.070 pictures posted within 50 meters of the buildings in our sample, with a large standard deviation of 46.25. Column 2 repeats the exercise for the subset of 999 buildings that were effectively rated by our sample respondents. One of the photos in our original survey sample of 1000 buildings was never randomly assigned to any rater; thus, our usable sample consists of 999 buildings. In columns 3 through 5 we separate the sample of buildings into those that have no pictures uploaded to them and those that have one or more photos in the 2011 vintage of Panoramio. This breakdown shows the strong correlation between image uploads in both Panoramio vintages and Flickr (2014).

**Table 1:** Summary statistics.

	Buildings by number of image uploads in Panoramio 2011					
	All Buildings		Buildings with no photos		Buildings with photos	
	*N* = 206,216	*N* = 999	*N* = 173,146	*N* = 607	*N* = 33,070	*N* = 392
	*Panel A. Image uploads*					
Panoramio image uploads within 50 meters (2014)	0.612 [4.156]	1.942 [5.813]	0.284 [2.634]	0.873 [4.348]	2.329 [8.240]	3.609 [7.249]
Panoramio image uploads within 50 meters (2011)	0.242 [0.652]	0.622 [0.970]	0.000 [0.000]	0.000 [0.000]	1.507 [0.862]	1.594 [0.928]
Flickr image uploads within 50 meters	2.070 [46.250]	3.751 [14.033]	1.585 [49.085]	2.873 [14.456]	4.610 [26.773]	5.121 [13.250]
Mean survey score		5.343 [1.083]		5.219 [1.090]		5.536 [1.045]
	*Panel B. Other Covariates*					
Building year	1954.57 [41.72]	1959.53 [70.21]	1959.04 [41.17]	1961.59 [85.43]	1933.69 [37.74]	1956.32 [35.02]
Building height	86.55 [88.30]	198.27 [133.51]	76.12 [74.40]	174.20 [113.24]	145.81 [128.53]	235.37 [152.70]

*Notes*: The table presents the sample means and standard deviation (in brackets) of the main variables used in our empirical analysis. Panel A summarizes image upload variables; Panel B summarizes other covariates. The first column summarizes the variables for our sample of 206,216 buildings; the second column shows the same statistics for the subsample of 999 that were rated by our survey respondents; the remaining columns present the summary statistics separately for buildings that had online images associated to them and those that were not geotagged with online pictures in the 2011 vintage of Panoramio (alternating again full and survey subsamples). Note that not all building characteristics are available for the whole sample.

**Fig 1:**
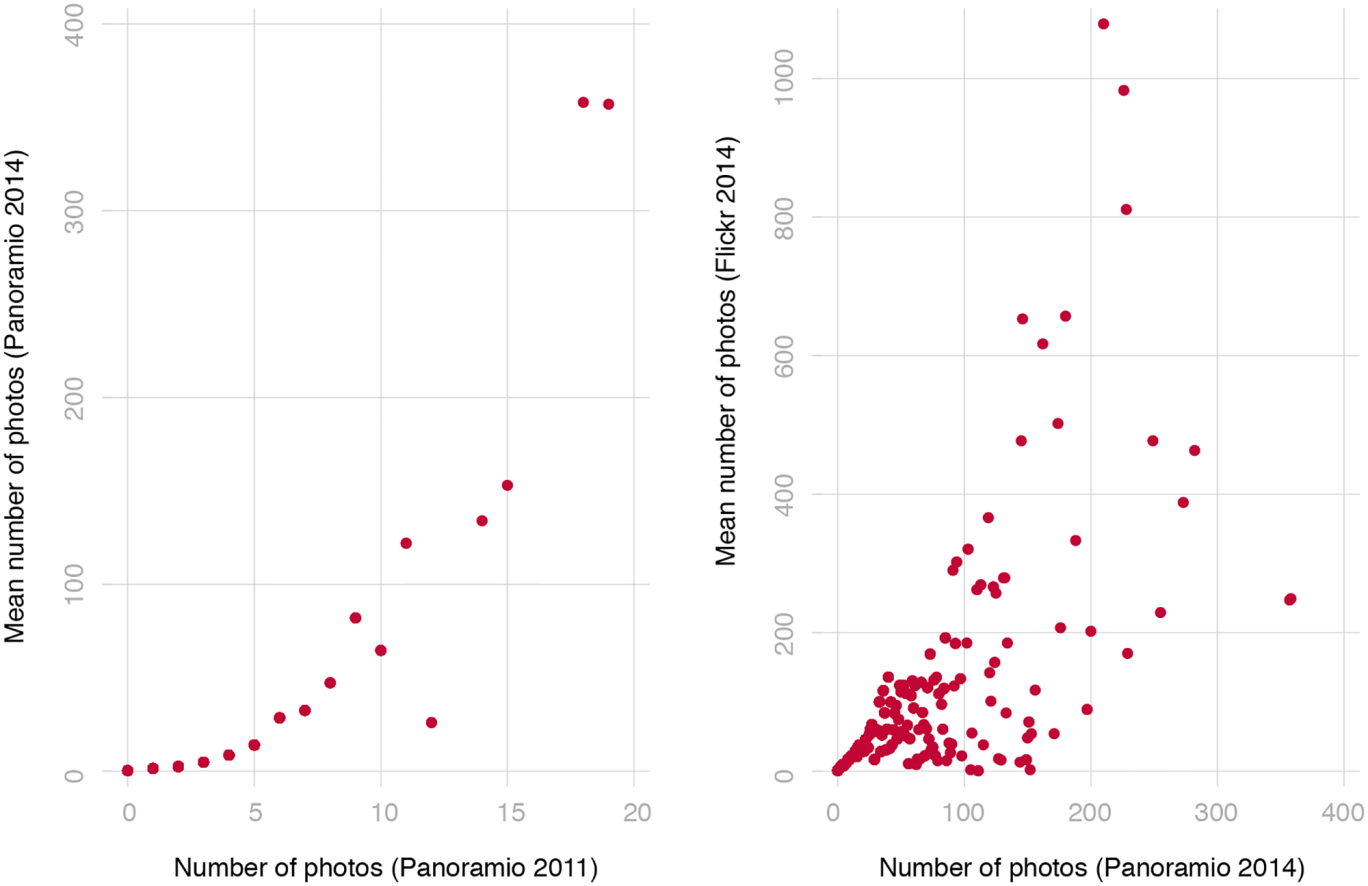
Relational scatter plots of the average number of photos within 50 meters 138 uploaded to the two Panoramio data vintages (left panel) and Panoramio and Flickr 139 (right panel).

Fig 1 plots the relationship between the number of photos uploaded to the two Panoramio vintages and Flickr. In Fig 1(a) we group buildings on the horizontal axis on the basis of the number of photos uploaded within 50 meters of them on Panoramio in 2011. Within each x-axis bin, we then calculate the average number of photos assigned by the 2014 vintage of Panoramio to the same buildings, which we plot in the y-axis. The pattern is consistent with photos that accumulate at an approximately proportional percentage rate, even though approximately 10 percent of the photos uploaded in 2011 were removed from the site by users in 2014. Buildings that had zero photos in 2011 had smaller chances of attracting large numbers of image uploads in 2014. Fig 1(b) plots the average number of photos uploaded to Flickr (vertical axis) by groups of buildings sorted according to the number of photos assigned using Panoramio’s 2014 vintage. Reassuringly, image upload measures are strongly correlated over time and across social media sites.

**Fig 2:**
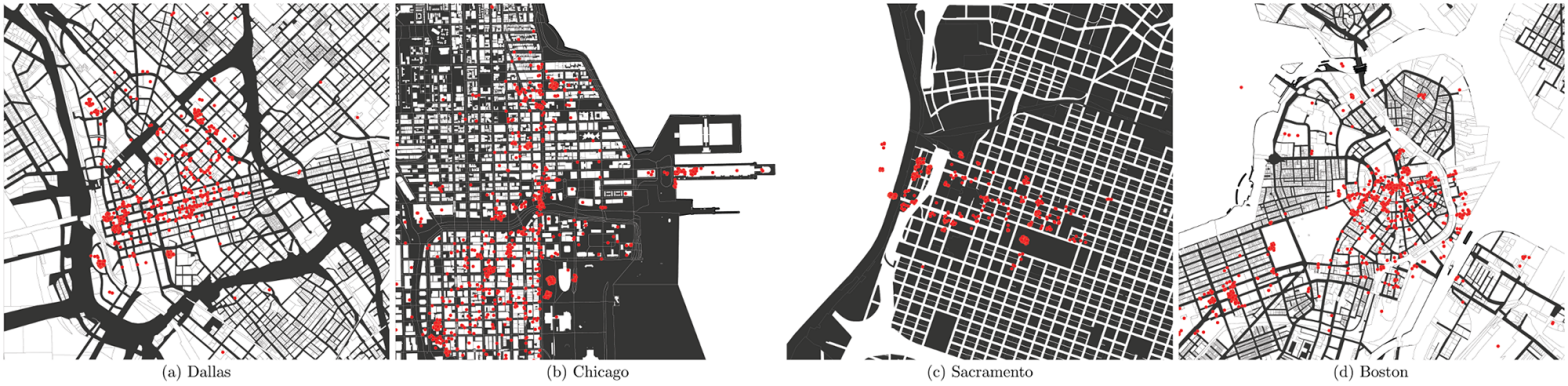
2014 image uploads within 50 meters of every building in four selected cities.

To illustrate the spatial distribution of our intended proxy for beauty value, we map the frequency of photos within a 50 meter radius of each building (image uploads) in the Emporis sample in four selected cities. As shown in Fig 2, despite a tendency for metropolitan centers and major cities to reveal overall higher number of uploaded photos, our maps show that less touristic cities, such as Sacramento and Dallas, also encounter a relatively high frequency of uploaded photos. Moreover, there is considerable variation in the distribution of photos within cities. S2 Fig in the appendix section maps the wide distribution of photo uploads throughout the U.S.

Having described how we created the frequency of image upload measurements for each building, we turn to the validation of such measurements as proxies for building beauty.

### Validation

Many factors may affect the propensity of internet users to upload photos. Some buildings acquire iconic status, regardless of their potential aesthetic appeal to observers who are unaware of their social importance. Others happen to be in high-traffic (e.g., touristic) areas or cities. Finally, internet users may upload photos for idiosyncratic reasons. Such reasons, which could amount to random noise in the data, need not invalidate the use of photo frequencies as proxies for average subjective beauty ratings. However, one can think of scenarios where: i) the noise to signal ratio is too high for image uploads to be practical; ii) idiosyncratic noise is correlated negatively with the latent variable of interest (e.g., if sharing photos of the most aesthetically unpleasant buildings was more entertaining to web users); iii) the aesthetic tastes of people actively sharing online content are substantially different from those of the public at large. Therefore, the use of geotagged image frequencies as proxies for the human appreciation of urban environmental features needs to be validated. To do this, we conducted a survey of the perception of beauty for a sub-sample of 1,000 buildings. The study (Protocol number: 1301005482) was reviewed and approved by the Committee on the Use of Humans as Experimental Subjects (COUHES) at MIT. We randomly selected buildings in the Emporis database in a way designed to *oversample those tagged with at least one internet upload* using the 2011 Panoramio vintage. Out of the initial 206,216 buildings, we settled on a sample size of 1,000, and randomly targeted to select 40 percent of the observations within the subsample of 33,070 buildings with any geotagged photo in 2011. Conversely, we targeted for the random selection of about 600 observations from the rest of our edifice population. Having a picture posted was a relatively rare occurrence; consequently, a powered research design required oversampling. In addition, the design allows for enough variance to trace the relationship between *the number* of photos taken for each building—not just whether a building has photos or not— and our survey-based measure of beauty. One of the randomly selected buildings happened to be the Empire State in New York City (an obviously iconic building with an outsized number of posted photos), for which we collected survey data, but which we have removed from the sample as an outlier. In a similar manner, we removed another two iconic buildings that were also found to be outliers (without substantial change to key results).

One could be concerned that a selection of images with more artistic flair might be more likely to be uploaded by internet users, and receive higher ratings independent of their intrinsic architectural value. Therefore, the stock images used in the survey were taken by professional photographers as provided by Emporis. 605 respondents ranked these buildings according to how beautiful they perceived them. The survey service was provided by Qualtrics^®^, conducted online, and attracted respondents from the 50 states in the U.S. and the District of Columbia, of which half reported living in metropolitan areas. Other collected demographic information included level of education, race/ethnicity, age, gender, and likelihood of posting public content on the internet. It is worth noting that average survey responses don’t vary significantly by age, gender, or location.

In the first stage of our survey, respondents were shown stock photographs of five distinct buildings (chosen randomly from three initial sets of five photos). They were then given up to 30 seconds to consider this set of photos side-by-side, which allowed them to get an idea of the range of the buildings that would be shown later in the survey. Providing these initial photo sets also allows us to have some consistency across respondents concerning the initial stimuli to which they were exposed, and it also makes it possible to test for potential statistical differences across starting frames, to which there were none. During the next 10 minutes, after seeing the five initial photographs side-by-side, respondents were shown a random sequence of single images of up to 1,000 buildings. To avoid framing effects [25], we did not provide any additional context to the photos. Respondents ranked each photo shown to them on a scale of 1 (ugliest) to 10 (most beautiful). After they had entered each image’s valuation or after 30 seconds (whichever occurred first), another photo appeared. We allowed participants to rank as many photos as possible during the survey’s 10-minute duration. To measure the consistency of respondents, two of the five initial practice photographs were shown to respondents again, which ensured that these pictures were ranked twice by each respondent.

Respondents provided an average of 107.76 image evaluations, rating one photo each 5.5 seconds. The maximum number of rated pictures provided by a single user was 365. Scientists still do not have a full understanding of the neural, affective, and cultural processes behind subjective beauty assessments [26]. However, the pace taken by even the fastest rater ensures that the two different types of brain networks involved in such judgments were likely engaged, since a 1.5 second interval is sufficient to activate both of them [27]. On the other hand, our design did not allow for very careful reflections on each image, since it has been shown that longer looking times are unlikely to change reported aesthetic preferences [28]. The mean survey score for ranked buildings was 5.342 and the standard deviation was 1.086.

To study whether buildings with higher image uploads in our survey were also considered more beautiful by respondents, we estimate the following baseline regression model:

$$\begin{array}{c} Score_{bi}=\alpha_{i}+\beta ImageUploads_{b}+\varepsilon_{b} \end{array}$$ (1)

Here *Score*_*bi*_ is the score that survey respondents *i* assigned to building *b*. *ImageUploads*_*b*_ is one of the measurements of the number of internet user-uploaded pictures geotagged at different distances from building *b*, and *β* is the coefficient of interest, which captures whether these measures are related to the subjective survey scores. Finally, *ε*_*b*_ is an error term. Since image uploads are building specific, we cluster standard errors at the building level.

The first panel in Table 2 displays OLS estimates of Eq (1) using 2014 Panoramio as the image uploads measure. Column 1 presents results using internet pictures geotagged within 50 meters of a given building; it includes no other controls or rater fixed effects. Our estimate implies that for every ten additional pictures, the average score obtained by a given building in our survey increased by 0.435 (standard error of the estimate = 0.103). Column 2 presents the most parsimonious specification with fixed effects for each of the 605 raters. Survey respondent dummies take account of systematic differences across individuals or in the environments under which the surveys were conducted. This specification also shows that for every ten additional pictures posted online, the average score obtained by a given building in our survey increased by 0.443 (standard error of the estimate = 0.102). To avoid framing effects, in column 3 we also control for a full set of dummies that capture the order in which a particular building appeared in each respondent’s session. In line with the random ordering of buildings in the survey, these controls have little impact on our estimates. In column 4 we address potential respondent inconsistency. By design, respondents were required to rank exactly 2 of the buildings twice during their session. For each respondent we compute the average absolute difference between rankings given to the same buildings. We then use the inverse of one plus the average differences to reweigh observations in the regressions. This procedure grants less weight to respondents who behaved inconsistently. Reassuringly, results remain unchanged.

**Table 2:** Estimates of the relationship between image uploads and building beauty: OLS and PCF estimates.

	Dependent variable: Average survey score				
	(1)	(2)	(3)	(4)	(5)
	*I. Panoramio photo uploads (2014)*				
Tens of photos within 0–50 meters	0.435*** (0.103)	0.443*** (0.102)	0.442*** (0.099)	0.464*** (0.104)	0.290*** (0.105)
Tens of photos within 50-100 meters					0.066 (0.056)
Tens of photos within 100-250 meters					0.014 (0.011)
Tens of photos within 250–500 meters					-0.003 (0.005)
Observations	65021	65021	65021	64507	65021
Clusters	996	996	996	996	996
R-squared	0.01	0.29	0.30	0.32	0.30
	*II. Flickr photo uploads*				
Tens of photos within 0–50 meters	0.125*** (0.037)	0.123*** (0.037)	0.123*** (0.036)	0.123*** (0.039)	0.107*** (0.041)
Tens of photos within 50-100 meters					0.015 (0.014)
Tens of photos within 100-250 meters					0.001 (0.003)
Tens of photos within 250–500 meters					-0.000 (0.003)
Observations	65021	65021	65021	64507	65021
Clusters	996	996	996	996	996
R-squared	0.00	0.29	0.30	0.31	0.30
	*III. Pcf photo uploads*				
Tens of photos within 0–50 meters	0.251*** (0.048)	0.252*** (0.048)	0.252*** (0.046)	0.258*** (0.049)	0.199*** (0.055)
Tens of photos within 50-100 meters					0.053 (0.060)
Tens of photos within 100-250 meters					0.057 (0.052)
Tens of photos within 250–500 meters					-0.028 (0.077)
Observations	65021	65021	65021	64507	65021
Clusters	996	996	996	996	996
R-squared	0.01	0.29	0.30	0.32	0.30
*Covariates and weighting*:					
Rater effects		✓	✓	✓	✓
Photo order effects			✓	✓	✓
Weighting by consistency (dif)				✓	

*Notes*: The number of photos within each annuli is shown in tens. The top two panels present OLS estimates of the relationship between image uploads and building beauty; the bottom panel presents PCF (principal Component Factor) estimates constructed using the common variation in Flickr and Panoramio 2014. The left-hand side variable is building beauty and the main explanatory variable image uploads. Observations are building and rater specific. Each column presents a different specification, and the bottom rows describe the covariates in each model. Below each of our estimates and in parentheses, we report standard errors that are robust to heteroskedasticity and clustered at the building level.

*** denotes a coefficient significant at the 1% level,

** at the 5% level, and

* at the 10% level.

In column 5 we augment our baseline specification by adding the number of pictures taken within 50-100, 100-250, and 250- 500 meters as controls. Estimates show that only pictures taken within 50 meters of a building can marginally predict the survey’s building beauty. The coefficients for the number of pictures taken further away are small, are close to zero, and are not statistically significant. These results suggest that image uploads in previous specifications were not proxying for broader neighborhood and regional differences but instead captured very localized effects.

**Fig 3:**
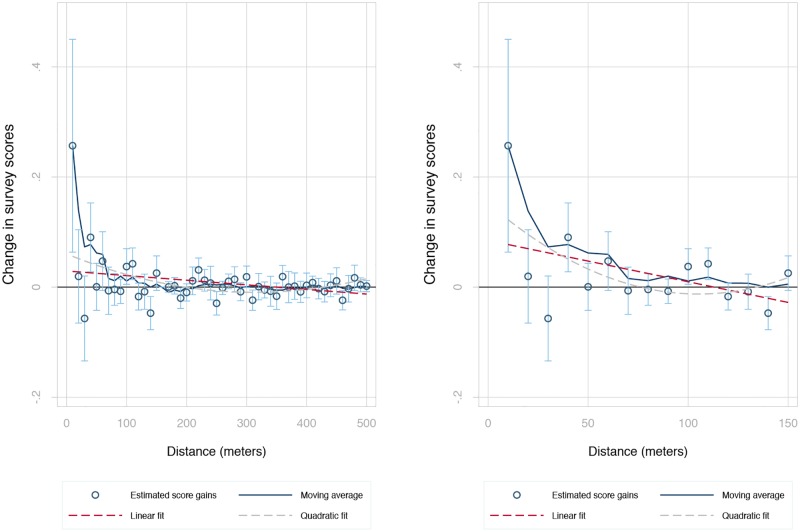
Estimates survey score marginal gains from pictures in range.

Fig 3 elaborates on these findings graphically using data from the 2014 vintage of Panoramio. In particular, we estimate similar models to those in the last column of Table 2, but allow pictures taken within each 10-meter range to take on a different coefficient. We then plot these coefficients and their confidence intervals, together with a smoothed moving average line and linear and quadratic fits. The left panel shows a marked decay in the information conveyed by pictures taken further away from a given building. Only the groups of pictures taken within 60 meters of a building predict its average score in our survey; while the remaining pictures have essentially zero additional predictive power. The right panel zooms in to illustrate the marked decay. S1 Fig in the online appendix displays a similar pattern using the Flickr data.

Panel B in Table 2 presents an analogous exercise to Panel A but uses Flickr photo uploads as the outcome of interest. Overall, we find a similar pattern as the one uncovered for photos uploaded to Panoramio. Column 3 shows that for every ten additional pictures, the average score obtained by a given building in our survey increases by 0.123 (standard error of the estimate = 0.036) and is statistically significant at the 1 percent level.

To exploit the common variation in Flickr and Panoramio, in Panel C we compute their principal component factor and use it as an alternative measure of image uploads. Column 3 shows that for an additional standard deviation in the number of photo uploads, the average score obtained by a given building in our survey increases by 0.252, which constitutes a substantial one-fifth of the standard deviation in building beauty across images.

### Coincident Covariation with Other Correlates of building beauty

Our previous results establish that buildings with higher image uploads also display stronger building beauty. We now explore whether observable physical characteristics of buildings drive part of this relationship. That is, do taller or more modern buildings consistently display higher building beauty? If so, for image upload frequency to be a good proxy, it should yield similar correlations. Alternatively, image uploads could capture unobserved dimensions of beauty beyond the measurable characteristics that are known to have an aesthetic impact.

We hence use the most commonly available building attributes in the Emporis database (height, year of construction, and dummies for architectural styles) as independent variables in an OLS regression in which we explain a building’s beauty as a linear function of these characteristics. This specification is available in Table Coincident Covariation with Other Correlates of building beauty of the online appendix section. The number of observations here corresponds to the number of buildings. Using the estimated coefficients of this regression, we separate building beauty as the sum of the component “predicted” by this OLS model and its “residual” component. In Table 3, column 1, we show results from another OLS regression using Panoramio image uploads 2014 as the dependent variable and the “predicted” component of building beauty as the main explanatory regressor. Reassuringly, a linear combination of building characteristics that are associated with higher building beauty is also a predictor of image uploads. Therefore, the image upload and building beauty metrics are not only correlated with each other; but they also covary in the same direction with observables, which is to be expected if they are capturing a similar phenomenon. Nevertheless, in column 2, we see that “residual” building beauty also correlates positively with image uploads, which suggests that the proxy contains information about beauty above and beyond that captured by the observed building characteristics that we were able to include in the model. For robustness, in columns 3 and 4 we also present negative binomial models that account for the discrete nature of the dependent variable. Reassuringly, we find similar results.

**Table 3:** Covariance between the predicted and residual components of building beauty and image uploads: OLS and Negative Binomial estimates.

	Dependent Variable: 2014 Panoramio Uploads (50 meters)			
	OLS		Negative Binomial	
	(1)	(2)	(3)	(4)
observable beauty	1.646*** (0.418)	1.737*** (0.423)	0.842*** (0.146)	0.855*** (0.135)
unobservable beauty		0.944*** (0.256)		0.389*** (0.082)
Observations	976	976	976	976
Clusters				
R-squared	0.03	0.05		

*Notes*: The table presents OLS and Negative Binomial estimates of regressions with Panoramio 2014 50-meter image uploads as the dependent variable. Independent variables are what we denominate “predicted” and “residual” beauty. Predicted beauty results from a linear combination of building characteristics (height, year of construction, and dummies for 26 architectural style types) fitted from OLS regression explaining building beauty. Residual beauty are the residuals from an OLS regression of building characteristics on building beauty. Each column presents a different specification. Below each of our estimates and in parentheses, we report standard errors that are robust to heteroskedasticity and clustered at the building level.

*** denotes a coefficient significant at the 1% level,

** at the 5% level, and

* at the 10% level.

### Image uploads predicts building beauty for demographic groups not active online

One potential concern of using image uploads as proxies for aesthetic value is that these measures could be capturing preferences exclusive to the population uploading content in photo-sharing websites. For instance, users of social media sites tend to be younger and more likely to live in urbanized areas. If planning boards used the covariates of image uploads to classify buildings “of interest”, such decisions might rely on a relatively narrow view of aesthetic quality. To address this concern, we asked survey respondents whether they had regularly uploaded content online. We now use this binary qualitative variable to estimate the following OLS model:

$$\begin{array}{cc} Score_{bi}= & \alpha_{i}+\beta_{N}ImageUploads_{b}\times Nonposter_{i}\\ & +\beta_{P}ImageUploads_{b}\times Poster_{i}+\varepsilon_{b}i. \end{array}$$ (2)

Here, *Poster*_*i*_ and *Nonposter*_*i*_ are dummy variables that indicate, respectively, whether respondent *i* reports regularly posting content online or not. 54.56 percent of the people in our sample are posters.

Columns 1 and 3 of Table 4 present the results of explaining building beauty using both Panoramio and -separately- Flickr image uploads interacted with the rater’s poster status. Again, standard errors are clustered at the building level. The coefficients for posters and non-posters in columns 1 and 3 are similar, which suggests that the frequency of image uploads and perception of beauty do not differ significantly for people with different posting habits. Of course, poster status is not a very fine-tuned variable for capturing differences across groups that have different online behaviors. Therefore, we next estimate a logit model that predicts the individual likelihood of posting content online based on demographic characteristics: gender, education, race, a dummy for metropolitan status, and age. We then estimate Eq [2], this time interacting image uploads with dummies for each of the quartiles of predicted poster likelihood (i.e., quartiles of the propensity score from the logit estimation) (*ImageUploads*_*b*_). Quartile 4 comprises the 25 percent of the survey respondents who have the highest estimated propensity score. Columns 2 and 4 show these estimates. In line with our previous results, the estimated coefficients for respondents in different quartiles are all similar and do not differ statistically from one another. This pattern suggests that image upload measures, crowdsourced exclusively from internet-posting users, are equivalently good predictors of building beauty for individuals who post less or no contents online.

**Table 4:** Estimates by demographic groups, panoramio 2014 and Flickr: OLS estimates.

	Dependent variable: Average survey score			
	Photos from Panoramio 2014		Photos from Flickr	
	By poster status	By likelihood of posting	By poster status	By likelihood of posting
	(1)	(2)	(3)	(4)
photos x non-posters	0.440*** (0.099)		0.133*** (0.032)	
photos x posters	0.445*** (0.102)		0.117*** (0.040)	
photos x quartile 1		0.442*** (0.103)		0.154*** (0.036)
photos x quartile 2		0.515*** (0.117)		0.158*** (0.039)
photos x quartile 3		0.476*** (0.101)		0.117*** (0.041)
photos x quartile 4		0.358*** (0.092)		0.073** (0.030)
Observations	64577	63251	64577	63251
Clusters	996	996	996	996
R-squared	0.30	0.29	0.29	0.29
*Covariates*:				
Rater effects	✓	✓	✓	✓
Photo order effects	✓	✓	✓	✓

*Notes*: The table presents OLS estimates of the demographic groups using Panoramio 2014 and Flickr. Each column presents a different specification, and the bottom rows describe the covariates and sample restrictions on each model. The Posters is an indicator for persons that responded “yes” to posting public content on the Internet for other people to use. Columns 1 and 3 present our results by poster status for Panoramio and Flickr, respectively; while columns 2 and 4 present our results by likelihood of posting. Below each of our estimates and in parentheses, we report standard errors that are robust against heteroskedasticity and clustered on buildings.

*** denotes a coefficient significant at the 1% level,

** at the 5% level, and

* at the 10% level.

## Discussion

Millions of internet users post content online. A growing scientific literature makes use of such online behavior to extract information about social trends. Here we have focused on websites that allow users to share images about the built environment. We have shown that the frequency at which photos are geotagged around a building predicts its average subjective beauty ratings. To validate this correlation we run a rating experiment with professional stock photos that are separate from online content.

The partial correlation between building beauty ratings and the frequency of image uploads posted around its geo-coordinates is strongest for images that are geotagged within 50 meters of a building’s address. Flickr users typically download the coordinates of the location where the photo was taken from their smart-phones, whereas Panoramio’s application makes it easier for users to locate the photo by identifying the feature at which the camera was pointing. Empirically, however, a 0–50 meter radius better captures image uploads as a proxy for building beauty for both data sets. Interesting differences between the two data sources in this regard are uncovered in the SI section.

The results validate the use of very localized image upload frequencies as an index to quantify architectural beauty for applications in architecture, urban planning, urban sciences, social psychology, and environmental studies. They therefore complement the existing literature on the use of online, geotagged user-generated content in those contexts (e.g. [21, 23, 24]).

Several current limitations of using image uploads as building beauty proxies are worth noting: i) The spatial distribution of uploaded photos is very skewed, with a large number of buildings in our sample of relatively notable ones displaying zero photos; image upload is therefore more likely to capture the right tail of the beauty distribution; ii) while predictive of average beauty survey ratings, most of the variance in subjective aesthetic evaluations between individuals and buildings remains to be explained; the use of image uploads is therefore not suitable for assessing or comparing specific buildings; it should therefore be constrained to statistical applications where the laws of large numbers apply; iii) if/as image upload measures become more popular, prospective use may be contaminated by strategic image uploads made to influence such measures; iv) other factors aside from beauty are also likely to impact image uploads; for instance, high pedestrian traffic or touristic areas are more likely to be pictured; note that such factors were made irrelevant in our research design due to the randomization of buildings and respondents in the survey, the anonymization of contextual building information (we did not provide information about city, neighborhood, or building names), and the focus on building beauty ratings for the buildings as such; nevertheless, confounding correlates of image uploads above and beyond building beauty will certainly be an issue in observational studies; good applications of image uploads as proxies for building beauty will therefore account for potential confounders (e.g., controlling for pedestrian traffic in a regression that tries to explain retail sales in buildings or city areas as a function of environmental image uploads).

The frequency of images assigned to each building using user volunteered geographic information will necessarily be a noisy measure of the actual images that refer to the specific buildings. This noise introduces a downward bias to our estimates of the relationship between image uploads and building beauty. In our particular setting this may be less of a problem because, despite the noise in our measures, the number of image uploads presumably preserves the relative ranking of buildings with respect to beauty. While we discuss this issue in more detail in the appendix, the fact that we obtain precise and positive estimates of the relationship between image uploads and building beauty suggests that there is enough signal in our measure to be of practical use to researchers.

On the other hand, some interesting issues are worth studying further: i) the issue of other potential correlates of image uploads is of intrinsic interest because it may lead to a better understanding of the behavioral patterns of internet users beyond their assessment of environmental beauty; ii) many applications of image uploads (like the extant [13] or [9]) use photo frequencies within larger geographical areas; it will be interesting to study the importance of contextual effects: is the sum of individual image uploads of buildings a worse or better statistic for the building beauty of larger areas? In order to better understand image uploads and building beauty dynamics across clusters, researchers could conduct similar rating surveys of urban environments using geographically-clustered images.

## Supporting information

S1 Fig Estimated survey score marginal gains from pictures in range (Flickr). (TIF)

S2 Fig Distribution of photo uploads throughout the U.S. (TIF)

S1 File Supplemental information. (PDF)
