# Supplementary figures and images for: Crowdsourcing architectural beauty: Online photo frequency predicts building aesthetic ratings
Source: PLoS One. 2018 Jul 25;13(7):e0194369. doi: 10.1371/journal.pone.0194369 (PMC6059390; doi:10.1371/journal.pone.0194369)

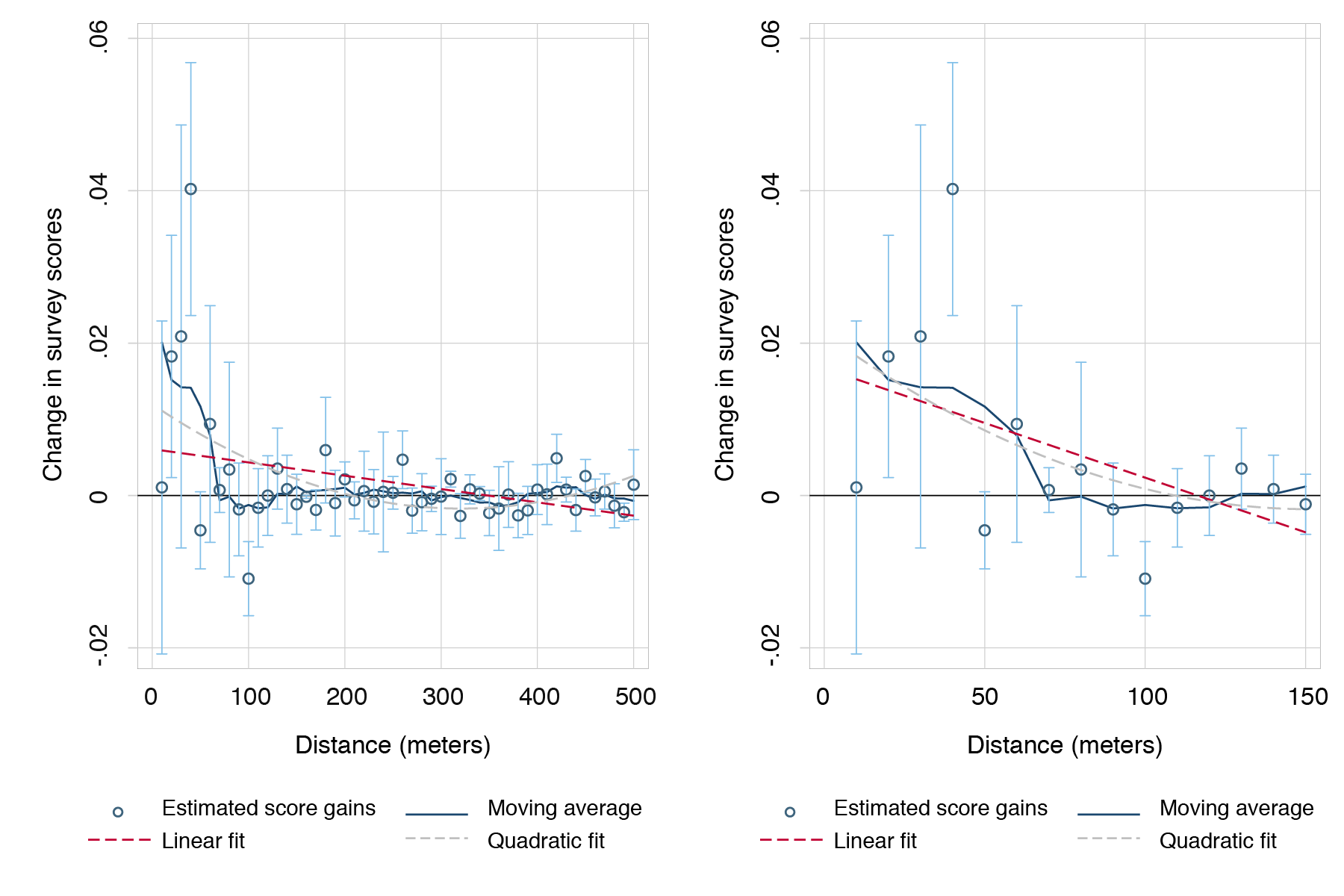

Supplement: S1 Fig — (TIF) [file pone.0194369.s001.tif]

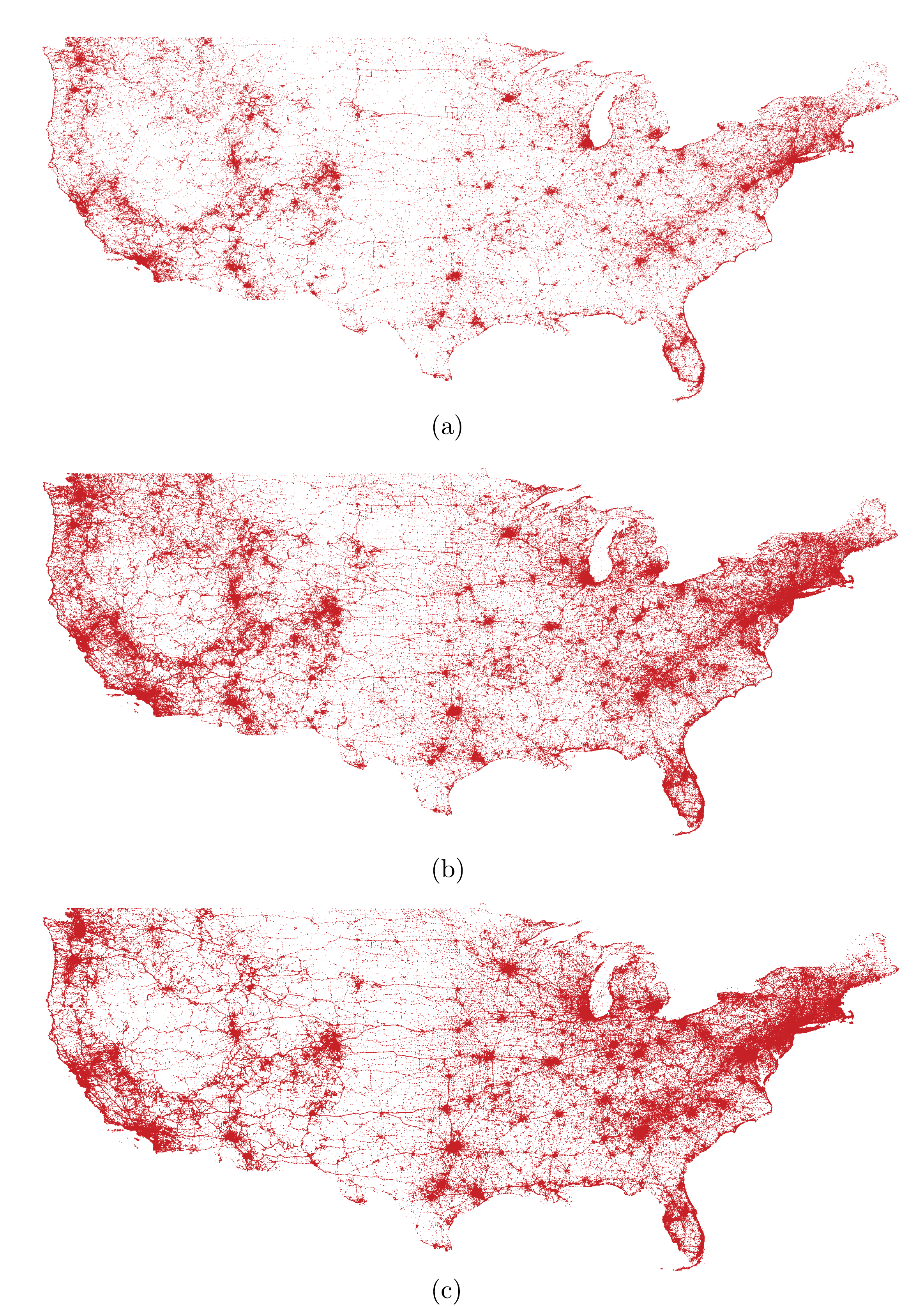

Supplement: S2 Fig — (TIF) [file pone.0194369.s002.tif]
