# Supplementary material for: Crowdsourcing architectural beauty: Online photo frequency predicts building aesthetic ratings
Source: PLoS One. 2018 Jul 25;13(7):e0194369. doi: 10.1371/journal.pone.0194369 (PMC6059390; doi:10.1371/journal.pone.0194369)
Supplement: S1 File — (PDF) [file pone.0194369.s003.pdf]

Online appendix for the paper "Crowdsourcing Architectural Beauty: Online Photo Frequency Predicts Building Aesthetic Ratings."

Albert Saiz* Arianna Salazar† James Bernard‡

This section contains supplementary material for the paper "Crowdsourcing Architectural Beauty: Online Photo Frequency Predicts Building Aesthetic Ratings." Section A1 discusses the differences between Panoramio and Flickr. Section A2 presents additional figures to illustrate the variation of image uploads. Section A3 shows additional robustness tests that validate the use of image uploads as a proxy for building beauty. Finally, in Section A4 we include a subset of the photos shown to the survey respondents (including their ranking) and a copy of the survey instrument.

A1 Panoramio, Flickr, and image uploads as Probability Fields: Potential Pitfalls, Promises, and Solutions

Both Panoramio and Flickr are examples of Volunteered Geographic Information (VGI) by internet users active on their websites (F. Goodchild (2007)). Therefore, we expect a degree of error in the measurement of the latitude and longitude in user-contributed geotagged photos. Many of these images contain precise coordinates

*Urban Studies and Planning Department, Massachusetts Institute of Technology, e-mail:saiz@mit.edu
†Urban Studies and Planning Department, Massachusetts Institute of Technology, e-mail: ariana@mit.edu
‡Economics Department, Brown University, e-mail: jamesbernard@brown.edu

from the Exchangeable Image File Format (Exif) used by cameras and smartphones with GPS systems. These coordinates capture the position from which the photo was taken. However, both Panoramio and Flickr allow users to pin down image locations on a digital map, absent Exif information. In both sources the pinning down of images to the position from which they were taken is encouraged by software, but users may be using alternative heuristics for photo location (et al. Larson (2015)). Nevertheless, since city street widths limit the distance between the position of the author and the landmark, we can expect for photos taken and uploaded around major urban buildings to have geotags that are proximate to the building's coordinates. Moreover, users taking photos at far distances from a building tend to geotag the objective.

Recent research by Zielstra and Hochmair (2013) has carefully analyzed the positional accuracy of images in Flickr and Panoramio.[1] These authors find Panoramio's geotags to be more accurate than Flickr's since its users are more likely to own better cameras and to be more sophisticated in geolocating their photos. Importantly, a large number of discrepancies are due to users geotagging the exact coordinates of the buildings they were capturing, not the point of where they were shooting (27 and 15 percent for North American street buildings in Flickr and Panoramio, respectively). Therefore, positional errors are generally biased in the direction of making photo geotags closer to the target buildings. Moreover, differences between Flickr and Panoramio can be attributed to the type of photo that is being posted. For example, Flickr photos tend to show a significant amount of human activity compared to Panoramio, which shows more scenic and landmark photography. Therefore, we expect Panoramio to be a better proxy for architectural beauty. In any case, the number of photos geotagged around each building needs to be considered probabilistically: more photos in the vicinity of a building signal a higher probability of that building being depicted. Type one classification errors (including pictures that are not about the building measured) and type two classification errors (missing pictures about a building that are posted beyond the distances that we explore in the paper) will typically bias down the relationship between image uploads and the actual building beauty metric. Therefore estimates in the article need to be interpreted as lower bounds for the quantitative impact of perfectly-measured image uploads

[1] Focusing on street buildings in the United States, Zielstra and Hochmair (2013) found the median distance between the camera position and the geotag of Flickr images to be of 31 meters. For Panoramio, the distance was only 15 meters.

on building beauty. Since researchers will tend to focus on the relative ranking by type of building rather than on the exact building beauty rating numbers (that is by definition designed by the survey creator), the downward bias may not be a problem in statistical applications that use image uploads under the laws of large numbers to capture building beauty across building types.

For example, consider two sets of different building types, which we conventionally denominate "brick" and "concrete." Assume that each set contains a vast and an identical number of buildings, but that the sum of the number of photos posted around "brick" buildings is double the number of photos posted around "concrete" buildings. Given the large number of observations, and if there are no reasons to assume that geotagging errors by internet users depend on building materials, the actual number of true photos of "brick" buildings should also be double (the rate of misclassification across groups should be constant in large samples). Furthermore, due to the results in the paper, it is likely for the beauty mean of the "brick" buildings to be substantially higher than the beauty mean for "concrete" buildings, which is sufficient for many -if not most- applications. Nevertheless, the differences in image uploads between the two groups multiplied by the coefficients reported in Table 2 of the main text are likely to underestimate their mean building beauty differences, due to attenuation bias.

Note that if researchers have a priori doubts about the randomness of measurement errors across categories (e.g. brick buildings tend to be in high-pedestrian traffic areas or narrower streets and therefore would get more type II misclassifications), then they should explicitly control for the potential sources of covariance that generate such measurement errors (e.g. control by pedestrian traffic or street width). In most applications, conditioning on neighborhood effects (e.g. zip code or census tract fixed effects) should control for localized differences in photo-taking behavior that could originate other patterns of misclassification error.

Another interesting and related issue has to do with the possible spatial correlation of building beauty. If architectural beauty tends to be geographically clustered, then type II classification errors (including photos from adjacent buildings) may be more likely for edifices within "beautiful" clusters. Beauty clustering may not be perceived as a problem for many applications if such clustering is systematic. In this case, the beauty of adjacent buildings can be thought of as an additional predictor of a building's beauty. In other applications, a straightforward way to bypass this issue is by including area fixed effects, so that we study differences in the impact of

building types within each cluster. However, our results in Table 2, Panel I, column 5, suggest that the additional information in annuli further away from the building (which are more likely to capture adjacent buildings) does not generate statistically significant coefficients. In other words, there does not seem to be a substantial contribution to explaining building beauty from photos that are more likely to be about neighboring buildings. Note that if neighboring beauty were adamant predictors of a building's beauty, we should have found stronger correlations. After all, 50-meter image uploads around one building is expected to be a noisier proxy for a buildings beauty than image uploads encompassing wider areas.

In conclusion, while building beauty may indeed be spatially correlated, local image uploads at 50 meter distances seem to be sufficient statistics for a building's beauty perceptions.

A2 Image uploads coverage

Figure A1 maps the location of all the photos posted to Flickr and Panoramio websites throughout the U.S. As mentioned in the main text, the total number of photos in 2011 Panoramio was approximately 800,000 and grew up to 3 million by 2014. The total number of photos posted to the Flickr website was approximately 13 million. The maps show how the photos are widely distributed across the U.S. The States with the highest number of photos per square kilometer uploaded to Panoramio 2014 are New Jersey (5.17), Rhode Island (4.63), and Massachusetts (3.41). For that same year, the States with the lowest number of photos per square kilometer are North Dakota (0.06), Kansas (0.09), and Mississippi (0.09).

A3 Robustness tests

This section presents additional robustness tests that validate the use of image uploads as a proxy for building beauty. As a first test, we present the scatter plots of the relationship between image uploads and building beauty. Figure A2 displays the graphic version of the relationship shown in Table 2 in the main text. On the vertical axis, we group buildings by the number of photos uploaded in their vicinity, measured using the 2011 and 2014 Panoramio, and Flickr websites. The vertical axis captures the sample mean building beauty ratings across buildings in our survey. The size of the dots are commensurate to the number of buildings within each

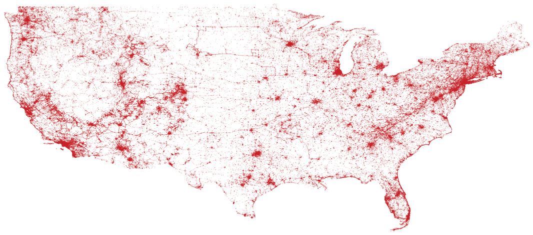

(a)

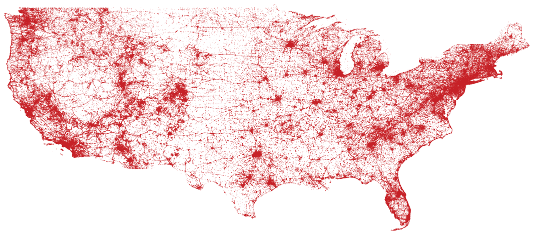

(b)

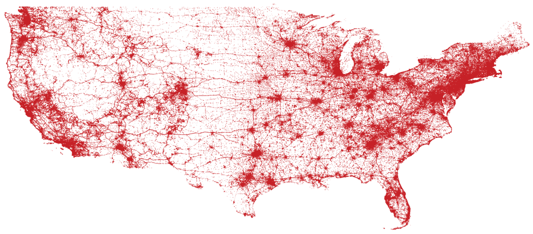

(c)

FIGURE A1: Map of all U.S. uploaded photos to Panoramio in 2011 (figure (a)), in 2014 (figure (b)), and Flickr (figure (c))

photo frequency bin. We display linear and quadratic fit lines. The figures show positive relationships between the number of uploaded photos and mean survey scores. Reassuringly, this positive relationship is not driven by outliers.

Our results in the paper show mostly pictures taken within 50-60 meters of a building in Panoramio can marginally predict the survey's building beauty. Figure A3 is analogous to Figure 3 in the main text, but uses Flickr photo uploads to illustrate the relationship. As in the paper, we run a regression with building beauty on the left hand side and a battery of variables capturing the number of photos within 10 meter-distance annuli (up to 500 meters) on the right hand side. The panel shows a marked decay in the marginal information conveyed by pictures taken further away from a given building. The right panel zooms in to further illustrate the marked decay. Our results are therefore robust to using different photo-sharing

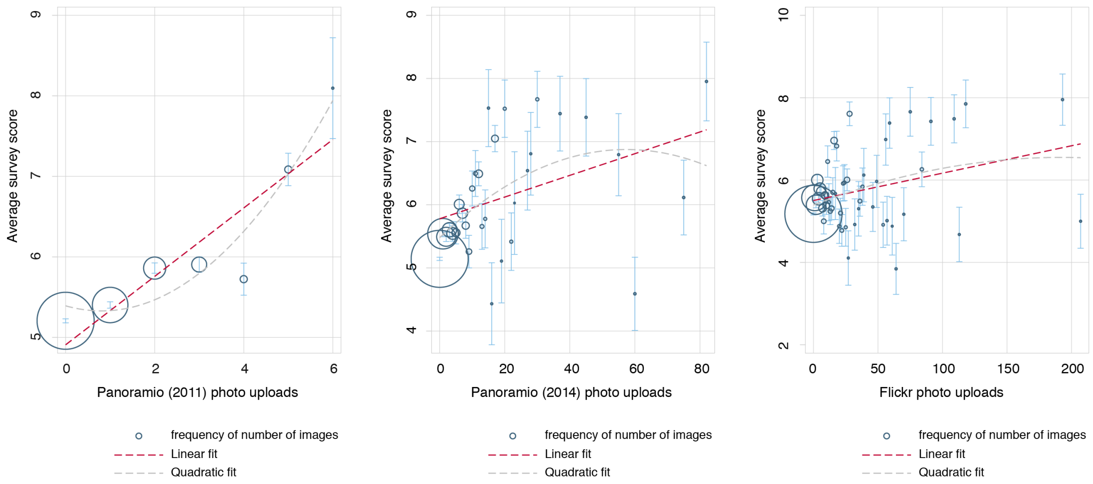

FIGURE A2: Scatter plots of the number of Panoramio photos in 2011 and 2014 against the mean survey score and Flickr photos.

websites. Interestingly, photo frequencies within the first 10 meters are less informative in Flickr. This could be consistent with the fact that Flickr users are more likely to upload coordinates directly from their phone, corresponding to the place from where they took the photo. Therefore, a setback of 10 meters would seem reasonable. Conversely, the Panoramio application makes it easy for users to pin photos to the exact geolocation of the buildings on a map. Alternatively, some of the differences in marginal significance between websites within the first 50 or 60 meters may just be random. Nevertheless, in both datasets, the sum of all photos at distances between 0 and 50 meters provides a strong predictor of building beauty, one that is strongly correlated across sites.

Table A1 shows the results of the regression that explains building beauty using height, age, and architectural style dummies, as used in Table 3 of the main text. We find that Spanish Colonial Revival and Beaux-Arts architectural styles receive on average a higher building beauty, while Modernist and Early Modernist buildings receive the lowest building beauty. [2] The fitted results from this regression (the explanatory variable values multiplied by the estimated coefficients) are used as our "predicted" component of beauty and the residuals (orthogonal to the explanatory variables) as our measure of "residual" beauty.

[2] The Spanish Colonial Revival Style is a United States architectural stylistic movement arising in the early 20th century based on the Spanish Colonial architecture of the Spanish colonization of the Americas. The Beaux-Arts style is known as a very rich, lavish and heavily ornamented classical style.

Table A1: Estimates of the relationship between observed characteristics, building beauty and image uploads measures: OLS estimates

	Average survey score
	(1)
building height	0.002***
	(0.000)
building year	0.005**
	(0.002)
Art Deco	0.496***
	(0.164)
Beaux-arts	0.748***
	(0.190)
Brutalism	-0.376
	(0.278)
Chateauesque	3.302***
	(0.965)
Chicago school	0.444
	(0.289)
Early modernism	0.234
	(0.280)
Gothic	3.488***
	(0.696)
International style	-0.049
	(0.175)
Neo-classicism	0.890***
	(0.170)
Neo-gothic	1.369***
	(0.235)
Postmodern	0.769***
	(0.097)
Renaissance revival	0.910***
	(0.205)
Romanesque revival	2.370***
	(0.309)
Spanish revival	0.537
	(0.354)
Observations	976
Clusters	
R-squared	0.24

Notes: The table shows the correlation between the observed architectural characteristics and building beauty. The table reports only the coefficients of the architectural dummies in the display to save space, but we include all characteristics in the regression. The omitted architectural style, which serves as a baseline is modernism. Below each of our estimates and in parentheses, we report standard errors that are robust against heteroskedasticity and clustered on buildings. *** denotes a coefficient significant at the 1% level, ** at the 5% level, and * at the 10% level.

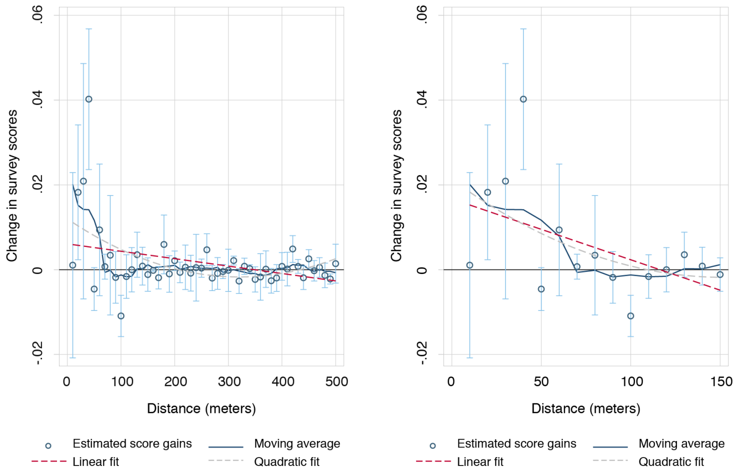

Figure A3: Estimated survey score marginal gains from pictures in range (Flickr)

As an additional robustness test we repeat the exercise in Table 2, Panel I, column 5 of the main text, this time allowing for the range of controls that we used in other columns in the same table. Table A2 presents the estimates of image uploads using two-dimensional annuli (”donuts”) of different lengths around each building in our sample. We then regress building beauty simultaneously against the number of photos uploaded in each annuli. The fact that the coefficients of photo uploads within 50 meters are robust across specifications confirms the highly localized nature of the relationship between image uploads and building beauty, which suggests that we are not confounding contextual factors, regional differences, or neighborhood effects.

A4 Respondents in Online Sample

As explained in the text, we used the services of a private vendor (Qualtrics) to conduct our survey online. We contracted an ex-ante random sampling of the US population, as opposed to much more expensive methods that explicitly stratify the sample to achieve the average population characteristics ex-post. In practice, this implies that we will have sampling error with regards to matching national characteristics. In addition, the procedure could also include errors introduced by the online sampling methods of the private vendors.

Heen et al. (2014) conducted an empirical examination of the composition errors of different primary survey providers: Survey Monkey, Qualtrics, and Mechanical Turk. On average, they find that online surveys tend to oversample the highly-

Table A2: Estimates of the relationship between photo uploads and survey scores, Panoramio 2014: OLS Estimates

	Dependent variable: Average survey score			
	(1)	(2)	(3)	(4)
	I. Photo count for all ratios			
Photos within 0-50 meters	0.264**	0.277**	0.290***	0.301***
	(0.108)	(0.108)	(0.105)	(0.110)
Photos within 50-100 meters	0.072	0.069	0.066	0.072
	(0.060)	(0.059)	(0.056)	(0.058)
Photos within 100-250 meters	0.016	0.017	0.014	0.015
	(0.012)	(0.012)	(0.011)	(0.012)
Photos within 250-500 meters	-0.003	-0.004	-0.003	-0.003
	(0.005)	(0.005)	(0.005)	(0.006)
Observations	65021	65021	65021	64507
Clusters	996	996	996	996
R-squared	0.01	0.30	0.30	0.32
Covariates and weighting:				
Rater effects		✓	✓	✓
Photo order effects			✓	✓
Weighting by consistency (dif)				✓

Notes: The number of photos within each annuli is shown in tens. Each column presents a different specification, and the bottom rows describe the covariates and sample restrictions on each model. Below each of our estimates and in parentheses, we report standard errors that are robust against heteroskedasticity and clustered on buildings. *** denotes a coefficient significant at the 1% level, ** at the 5% level, and * at the 10% level.

educated and white, but do well in other dimensions. In general, they conclude that "for many applications, the advantages of online surveys (e.g., the efficiency of data collection, lower economic costs, and acceptable approximations to population profiles) far exceed their disadvantages regarding external validity."

Table A3 reports the demographic characteristics of our survey conducted in 2013 and the American Community Survey (ACS) 5-year from 2009 to 2013. Among the demographic characteristics, we have age reported in brackets (under 20, 20-30, 30-40, 40-50, and 50 or older) and gender. Our survey also includes the race of the respondent, which corresponds to one of the following categories: White/non-Hispanic, African American, Asian, Hispanic, and Other. Also, the survey reports the education level of respondents, which varies from high-school graduates to respondents with some college or completed college and respondents with less than high school. Finally, concerning geography, our survey reports whether respondents live in a metropolitan area and their state of residence.

Some of the survey characteristics do not deviate much from those reported in the census. However, as in Heen et al. (2014), the online survey did tend to oversample whites. The survey's largest discrepancy is with regards to metropolitan area status. The frequency of self-reported metropolitan status is much smaller than we would expect based on random sampling. Such discrepancies could arise from different conceptions about how a metropolitan area is perceived by respondents. However, we take the discrepancies at face value to assess the robustness of the findings.

In order to see if the existing differences between the census sample and survey in table A3 impact the covariance between assessed beauty and online photo frequencies we conduct additional exercises to reweigh the survey data to match the frequency of the census demographic categories. In these exercises, we eliminate the respondents who report "unknown" in the survey (amounting to 18 people). The first row of table A4 includes gender and age. Results are virtually identical to the ones in table 2 of the main text. In the following columns, we continue adding additional variables (race, education, state, and finally metro area) and reassuringly our results remain the same as those in Table 2.

When we attempt to match the frequency of a few categories (for instance age and gender) in the first row of table A4, we can do it perfectly. Naturally, as we increase the number of categories the census bins become more sparse, and we cannot perfectly match their frequency. For instance, in the last row of table A4, we have about 15,000 census bins and a sample of 586 individuals. Nevertheless, reweighing

Table A3: Census and Survey Comparison

		Percentage of Individuals	
		Survey	(ACS) 5-year
Gender	Unknown	0.71%	
		[0.46%-0.95%]	
	Male	48.31%	48.57%
		[46.84%-49.79%]	
	Female	50.98%	51.43%
		[49.50%-52.46%]	
Age	Unknown	0.43	
		[0.24%-0.63%]	
	50 and older	36.77	42.86
		[35.34%-38.19%]	
	40 - 50	17.08	18.17
		[15.97%-18.20%]	
	30 - 40	18.86	16.98
		[17.70%-20.02%]	
	20 - 30	22.30	18.21
		[21.07%-23.53%]	
	Under 20	4.56	3.77
		[3.94%-5.17%]	
Race	Unknown	0.57	
		[0.35%-0.79%]	
	White, Non-Hispanic	79.2	66.42
		[78.00%-80.40%]	
	African American	6.77	12.02
		[6.02%-7.51%]	
	Asian	5.63	5.18
		[4.94%-6.31%]	
	Hispanic	5.15	9.44
		[4.49%-5.80%]	
	Other	2.69	6.94
		[2.21%-3.17%]	
Metro Area	Unknown	2.19	9.75
		[1.75%-2.62%]	
	Yes	48.41	77.36
		[46.93%-49.88%]	
	No	49.41	12.89
		[47.93%-50.89%]	
Education Level	Unknown	0.89	
		[0.61%-1.17%]	
	High School Degree	42.39	37.04
		[40.93%-43.85%]	
	College Degree or Higher	50.05	50.57
		[48.54%-51.50%]	
	Other	6.7	12.39
		[5.96%-7.44%]	

Notes: The table presents the percentage of respondents in the survey and the American Community Survey (ACS) 5-year from 2009 to 2013 broken down by their demographic and geographic characteristics.

does eliminate *expected* biases in the relative frequencies of characteristics.

As stated, concerns about sample composition turn out not to be relevant in practice: our estimates remain extremely robust with all alternative weight rebalancing schemes. In the bottom panel of Table A4, we lose an additional survey respondent (hence the decline in observations) for which we cannot find its corresponding weights in the ACS sample. That is, there no respondent in the ACS with the same demographic characteristics.

For instance, we can compare the coefficients in the bottom panel, where we thoroughly reweigh using census frequencies concerning all variables in the sample. The coefficients of the impact of survey scores on photo frequencies are 0.447, 0.128, and 0.261 in Panoramio, Flickr, and their first principal component factor, respectively. These are comparable to 0.442, 0.123, and 0.252 in the unweighted counterpart results in Table 2, column (3) of the main text. In the worst-case scenario, the differences are below one-sixth of the estimated standard deviation of the parameter.

We conclude that –in our case– the raw online sample provides researchers with valid variation for investigating issues related to the environmental covariates of perceived architectural beauty. Moreover, the use of similar online rating exercises can provide researchers with a cost-effective way to study architectural beauty in other contexts as well. Conducting off-line image ratings with significant respondent samples (e.g., over 500) and many image ratings (exceeding 100 per respondent), might make these exercises prohibitively expensive, thereby curtailing such investigations. Re-weighting exercises –as we do here– can then be conducted to assess the robustness of results to sampling conditions.

Table A4: Main results reweighing by survey characteristics

	Dependent variable: Average survey score		
	(1)	(2)	(3)
	Panoramio photo uploads	*Flickr photo uploads*	*Pcf photo uploads*
Reweighting by gender and age	0.446***	0.128***	0.259***
	(0.103)	(0.037)	(0.047)
Observations	63251	63251	63251
Clusters	996	996	996
R-squared	0.29	0.29	0.29
Reweighting by gender, age,	0.447***	0.122***	0.254***
and race	(0.100)	(0.036)	(0.047)
Observations	63251	63251	63251
Clusters	996	996	996
R-squared	0.29	0.28	0.29
Reweighting by gender, age,	0.440***	0.125***	0.254***
race, and education	(0.099)	(0.036)	(0.048)
Observations	63251	63251	63251
Clusters	996	996	996
R-squared	0.29	0.29	0.29
Reweighting by gender, age,	0.445***	0.131***	0.262***
race, education, and state	(0.110)	(0.039)	(0.051)
Observations	63251	63251	63251
Clusters	996	996	996
R-squared	0.28	0.28	0.28
Reweighting by gender, age, race,	0.447***	0.128***	0.261***
education, state, and metro area	(0.116)	(0.042)	(0.054)
Observations	63207	63207	63207
Clusters	996	996	996
R-squared	0.30	0.29	0.30
Covariates:			
Rater effects	✓	✓	✓
Photo order effects	✓	✓	✓

Notes: The dependent variable is average survey score. Observations are building and rater specific. Each column presents a different specification, where we vary the source of image uploads. The bottom rows describe the covariates in each model. Below each of our estimates and in parentheses, we report standard errors that are robust to heteroskedasticity and clustered at the building level. *** denotes a coefficient significant at the 1% level, ** at the 5% level, and * at the 10% level.

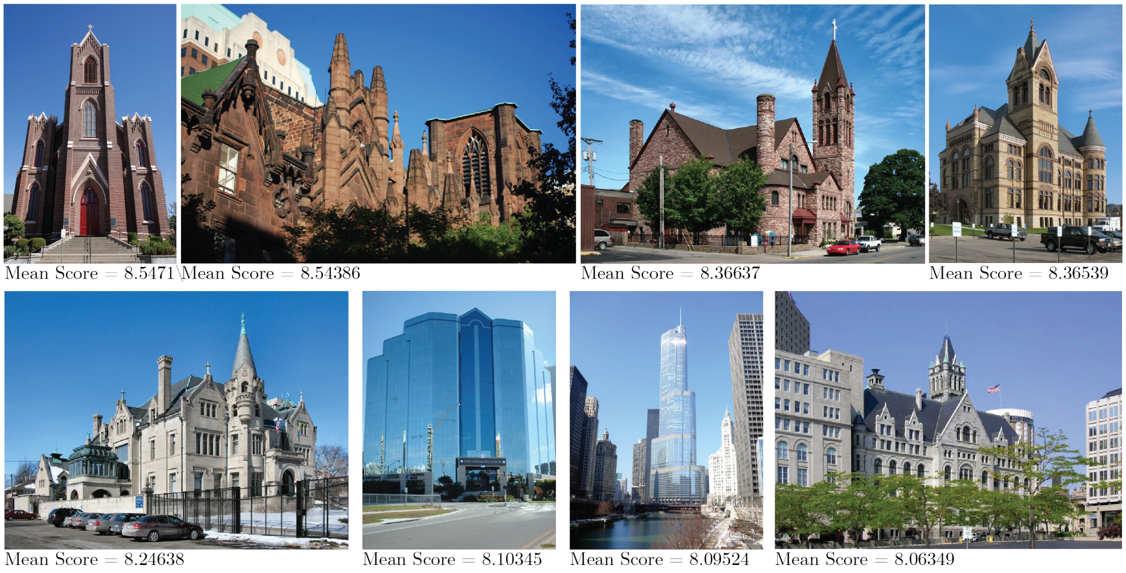

FIGURE A4: Top survey photos ranked by mean respondent scores.

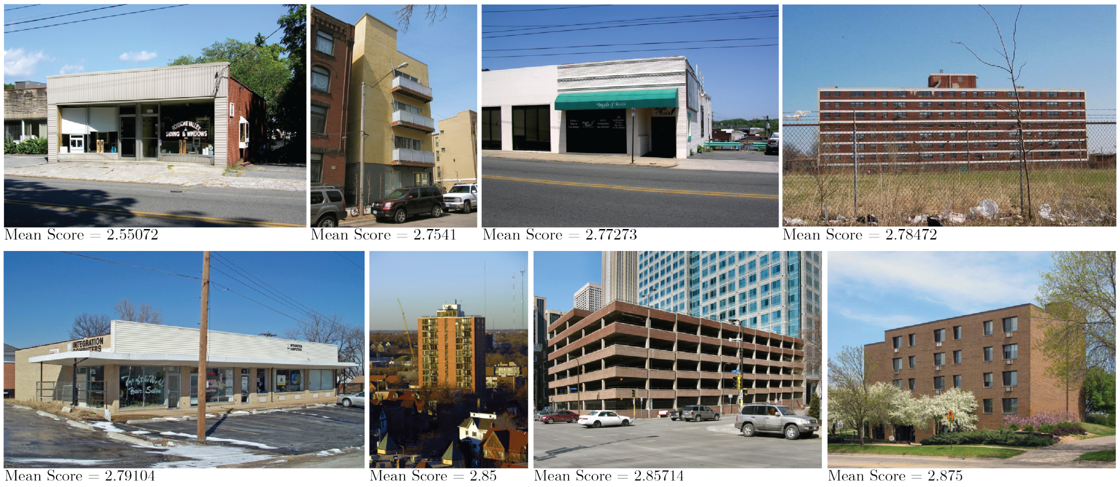

FIGURE A5: Bottom survey photos ranked by mean respondent scores.

References

et al. Larson, M. (2015). *The Benchmark as a Research Catalyst: Charting the Progress of Geo-prediction for Social Multimedia.*

F. Goodchild, M. (2007). Citizens as Sensors: The World of Volunteered Geography. *GeoJournal*, 69:211–221.

Heen, M. S. J., Lieberman, J. D., and Miethe, T. D. (2014). A Comparison of Different Online Sampling Approaches for Generating National Samples. *Ccjp*, 1(September):1–8.

Zielstra, D. and Hochmair, H. H. (2013). Positional accuracy analysis of Flickr and Panoramio images for selected world regions. *Journal of Spatial Science*, 58(2):251–273.

Survey Instrument

What Is Your Gender?

- Male
- Female

What Is Your Age

- 50 and older
- 40-50
- 30-40
- 20-30
- Under 20

What Is Your Race/Ethnicity?

- White, non-Hispanic
- African American
- Asian
- Hispanic
- Other

What State do You Live in?

Pennsylvania

Do you live in a metropolitan area?

- Yes
- No

What level of education do you have?

- High School degree
- College degree or higher
- Other

Have you ever posted some kind of public content on the internet for other people to use anonymously (excluding content available to your family and friends only), such as photos, blogs, spreadsheets, movie or restaurant reviews, product descriptions, etc?

- Yes
- No

In a typical month, how often do you post public content on the internet for other people to use anonymously?

Please answer the question

Continue

You will now be shown five photographs of buildings. Please take 20-30 seconds to consider the photographs, and think about how you would rate these buildings on a scale from 1 (ugliest) to 10 (most beautiful). After you are done looking at them, you will be shown each photo individually and asked to rate each photo. When you are ready to begin, please hit the continue button below.

Continue

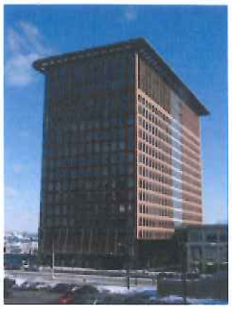
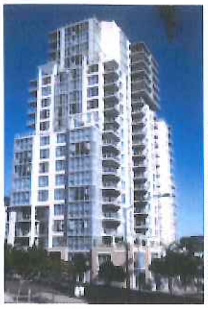
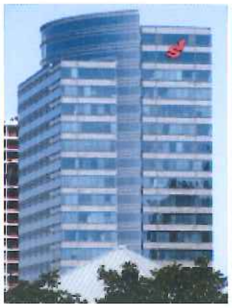
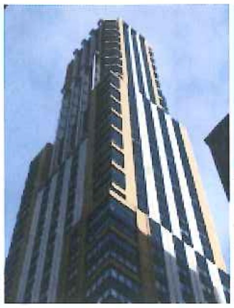
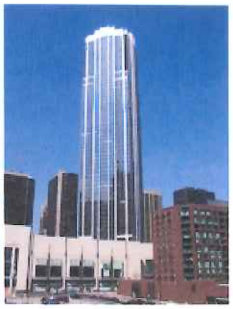

Continue

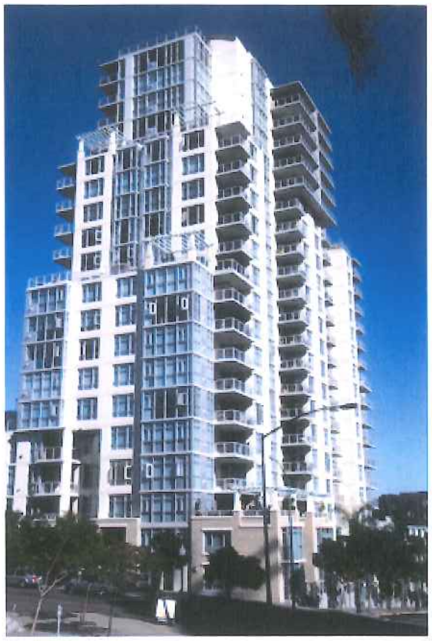

Please evaluate the most prominent building in the picture on a scale from 1 (ugliest) to 10 (most beautiful).

1 2 3 4 5 6 7 8 9 10

Continue

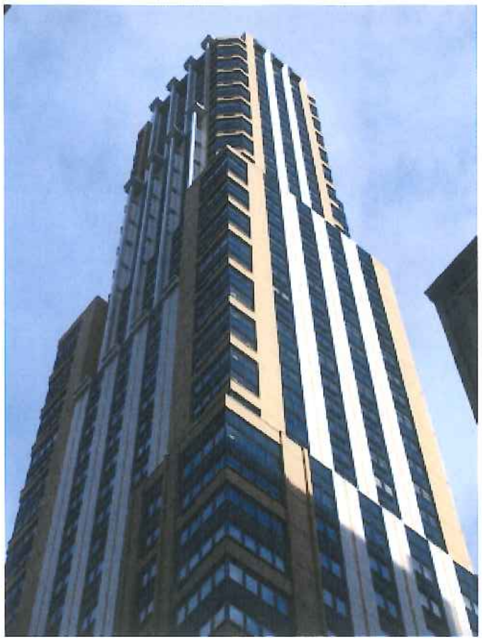

Please evaluate the most prominent building in the picture on a scale from 1 (ugliest) to 10 (most beautiful).

1	2	3	4	5	6	7	8	9	10
○	○	○	○	○	○	○	○	○	○

Continue

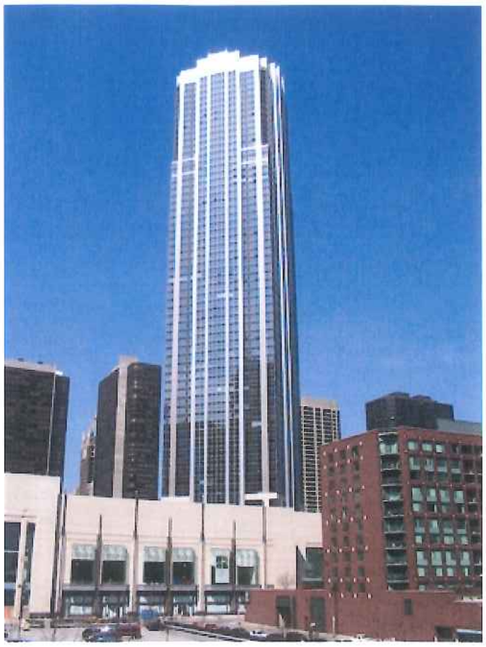

Please evaluate the most prominent building in the picture on a scale from 1 (ugliest) to 10 (most beautiful).

1	2	3	4	5	6	7	8	9	10
○	○	○	○	○	○	○	○	○	○

Continue

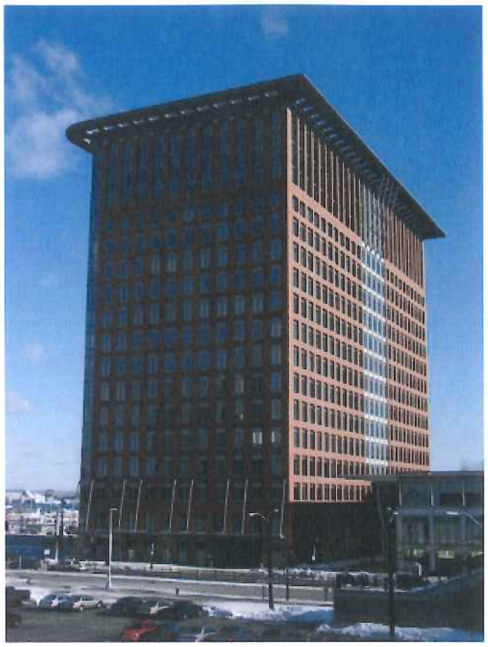

Please evaluate the most prominent building in the picture on a scale from 1 (ugliest) to 10 (most beautiful).

1	2	3	4	5	6	7	8	9	10
○	○	○	○	○	○	○	○	○	○

Continue

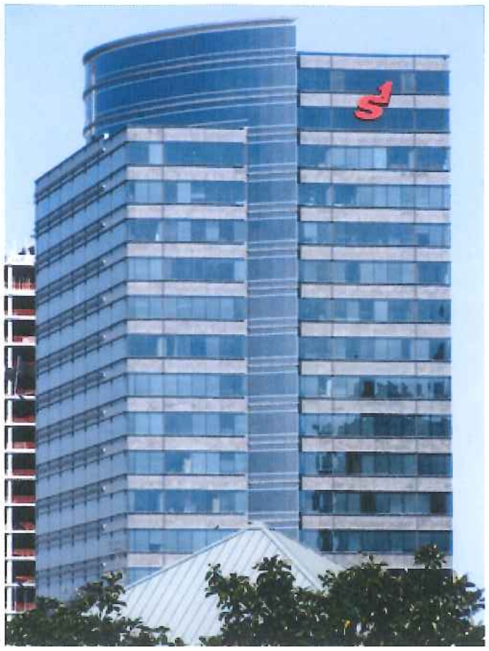

Please evaluate the most prominent building in the picture on a scale from 1 (ugliest) to 10 (most beautiful).

1 2 3 4 5 6 7 8 9 10

Continue

You will now be asked to rate those five buildings on a scale from 1 (ugliest) to 10 (most beautiful). When you are ready to begin, please hit the continue button below.

Continue

Showing Photo 5

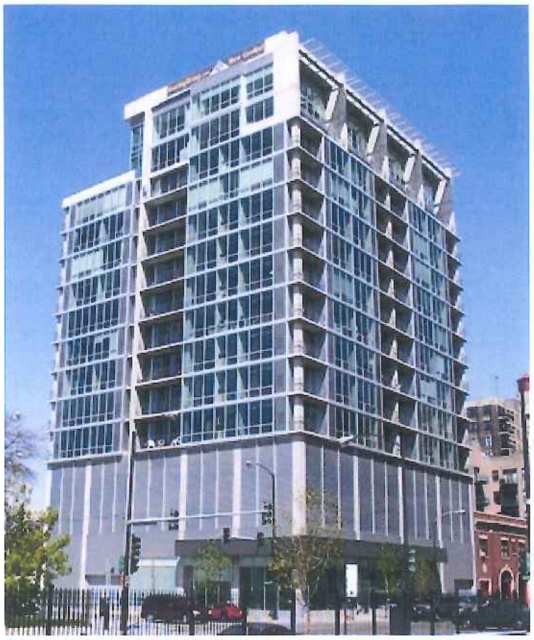

Please evaluate the most prominent building in the picture on a scale from 1 (ugliest) to 10 (most beautiful).

1	2	3	4	5	6	7	8	9	10
○	○	○	○	○	○	○	○	○	○

Continue

Showing Photo 6

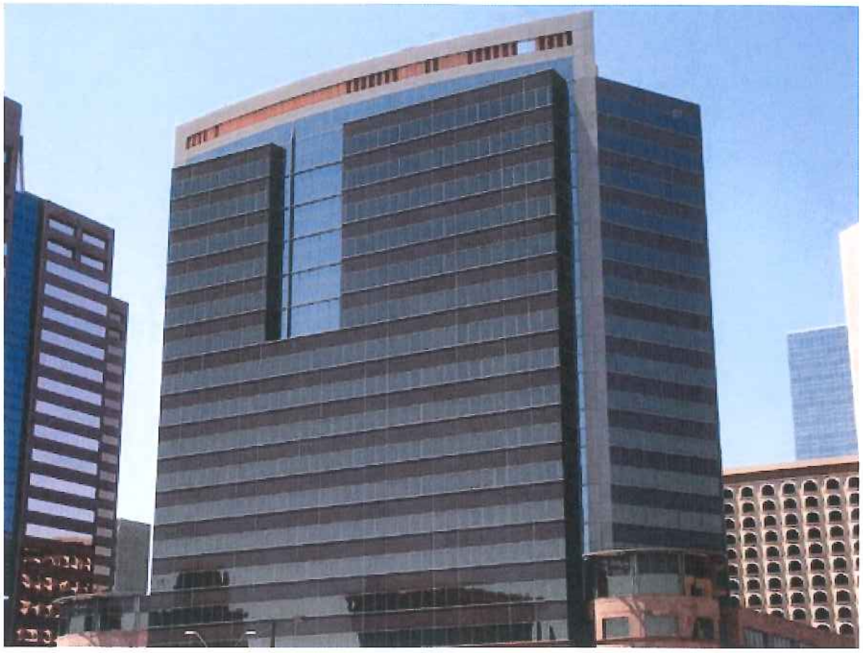

Please evaluate the most prominent building in the picture on a scale from 1 (ugliest) to 10 (most beautiful).

1	2	3	4	5	6	7	8	9	10
○	○	○	○	○	○	○	○	○	○

Continue

Showing Photo 8

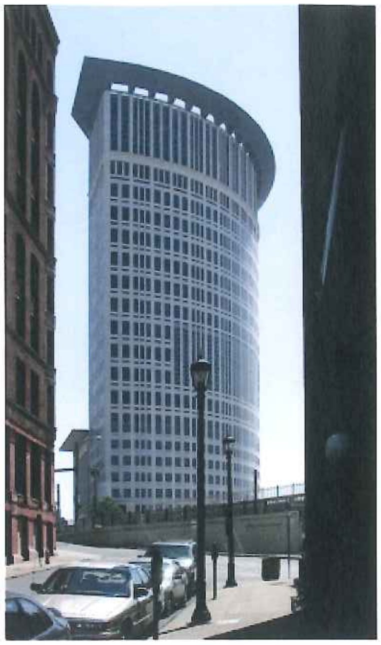

Please evaluate the most prominent building in the picture on a scale from 1 (ugliest) to 10 (most beautiful).

1	2	3	4	5	6	7	8	9	10
○	○	○	○	○	○	○	○	○	○

Continue
